# Gene cloning and characterization of a novel esterase from activated sludge metagenome

**DOI:** 10.1186/1475-2859-8-67

**Published:** 2009-12-22

**Authors:** Tao Zhang, Wen-Jun Han, Zhi-Pei Liu

**Affiliations:** 1 State Key Laboratory of Microbial Resources, Institute of Microbiology, Chinese Academy of Sciences, Beijing 100101, PR China; 2 School of Life Sciences and Biotechnology, Mianyang Normal University, Mianyang 621000, PR China

## Abstract

A metagenomic library was prepared using pCC2FOS vector containing about 3.0 Gbp of community DNA from the microbial assemblage of activated sludge. Screening of a part of the un-amplified library resulted in the finding of 1 unique lipolytic clone capable of hydrolyzing tributyrin, in which an esterase gene was identified. This esterase/lipase gene consists of 834 bp and encodes a polypeptide (designated EstAS) of 277 amino acid residuals with a molecular mass of 31 kDa. Sequence analysis indicated that it showed 33% and 31% amino acid identity to esterase/lipase from *Gemmata obscuriglobus *UQM 2246 (ZP_02733109) and *Yarrowia lipolytica *CLIB122 (XP_504639), respectively; and several conserved regions were identified, including the putative active site, HSMGG, a catalytic triad (Ser92, His125 and Asp216) and a LHYFRG conserved motif. The EstAS was overexpressed, purified and shown to hydrolyse *p*-nitrophenyl (NP) esters of fatty acids with short chain lengths (≤ C8). This EstAS had optimal temperature and pH at 35°C and 9.0, respectively, by hydrolysis of *p*-NP hexanoate. It also exhibited the same level of stability over wide temperature and pH ranges and in the presence of metal ions or detergents. The high level of stability of esterase EstAS with its unique substrate specificities make itself highly useful for biotechnological applications.

## Introduction

Lipolytic enzymes such as esterases (EC3.1.1.1) and lipases (EC3.1.1.3) catalyze both the fat hydrolysis and the synthesis of fatty acid esters including acylglycerides as biocatalysts [1]. Lipolytic enzymes are ubiquitous α/β hydrolyzing enzymes existed in animals, plants, and microbes, including fungi and bacteria. Microbial esterases are showing considerable industrial potential where their regiospecificity and enantioselectivity are desired characteristics [2], such as production of fine chemicals, pharmaceuticals, in the food industry and are widely used in biotechnology [2-4].

Modern biotechnology has a steadily increasing demand for novel biocatalysts, thereby prompting the development of novel experimental approaches to find and identify novel biocatalyst-encoding genes. Metagenome is the total microbial genome directly isolated from natural environments, and the power of metagenomics is the access, without prior sequence information, to the so far uncultured majority, which is estimated to be more than 99% of the prokaryotic organisms [5-7]. In fact, the metagenomic approach was successful in searching for novel lipolytic enzymes in varied environments, and also, several genes encoding metagenomic esterases have been identified in metagenomic libraries prepared from varied environmental samples, including soils [6-9], marine sediment [10-12], pond and lake water [13-15], and tidal flat sediment [16].

Studies based on 16S rDNA library have extensively redefined and expanded our knowledge of microbial diversity in activated sludge from low-temperature aromatic wastewater treatment bioreactor, including members of various un-culturable groups (unpublished data). To the best of our knowledge, activated sludge microbial communities have not been exploited by culture-independent methods for isolation of lipolytic genes. Here, we report the isolation, sequence analysis, and enzymatic characterization of a novel esterase, EstAS, from an activated sludge derived metagenomic library. The discovery of EstAS led to the identification of a new family of bacterial lipolytic enzymes.

## Materials and methods

### Sampling

Activated sludge was collected from a low temperature sequencing batch bioreactor (SBR) treating nitrogen-containing aromatic wastewater in our laboratory.

### Bacterial strains, plasmids, and growth conditions

The starting strains and plasmids used in this study are listed in Table 1. *E. coli *was grown at 37°C in Luria-Bertani (LB) medium supplemented with appropriate antibiotics [17], at 12.5 μg/ml for chloramphenical, 100 μg/ml for ampicillin and 25 μg/ml for kanamycin.

**Table 1:** Bacterial strains and plasmids used in this study

Strain or plasmid	Description	Source or reference
**Strains**		
*E. coli *EPI300™-T1R	*[F- e14-(McrA-) D(mcrC-mrr) (TetR) hsdR514 supE44 supF58 lacY1 or D(lacIZY)6 galK2 galT22 metB1 trpR55 l-]*	Epicentre
*E. coli *TOP10	*lac*x*74 recA1 deoR F - mcrA*Δ (*mrr-hsdRMS-mcrBC*) *φ80 lacZ*Δ*M15*Δ *araD139*Δ *(ara-leu)7697 galU galK*	TianGen
*E. coli *BL21(DE3)	*F-, ompT, hsdSB (rB-, mB-), dcm, gal, λ(DE3), pLysS, Cmr*	Novogen
*E. coli *EPI300-FosB12L1	Positive clone from Fosmid genomic library, which carries the lipolytic gene	This study
*E. coli *TOP10-EstAS	Positive clone from sublibrary, which carries the *EstAS *gene fragment	This study
*E. coli *BL21(DE3)-EstAS	Positive clone, which carries the pEstAS-His expression vector	This study
**Plasmids**		
pCC2FOS	Cloning vector; Cm^r^	Epicentre
pUC118	Cloning vector; Ap^r^	Takara
pET28a	Expression vector; Km^r^	Novagen
FosB12L1	pCC2FOS, which carries the *EstAS *gene cluster (35 kb)	This study
EstAS	pUC118, which carries the complete lipolytic gene (*EstAS*)	This study
pEstAS-His	pET28a carrying amplified *Hin *dIII -*Nde *I fragment containing lipolytic gene (*EstAS*)	This study

Ap^r^, ampicillin resistant; Cm^r^, chloramphenicol resistant; Km^r^, kanamycin resistant.

### DNA preparation and manipulation

*E. coli *cells were transformed by the calcium chloride procedure [17]. Recombinant plasmid DNA was isolated by the method of Birnboim and Doly [18] or with a Tian-prep Mini kit (TianGen). Restriction enzymes, T4 DNA ligase and calf intestinal alkaline phosphatases were purchased from New England Biolabs (Ipswich, USA) or Takara (Tokyo, Japan) and used according to the manufacturers' instructions.

### Construction of metagenomic DNA library and sublibrary

Activated sludge DNA extraction was carried out using SDS and proteinase K treatment [19], and the removal of humic acids (HAs) prior to DNA extraction was conducted by using HAs removing buffer [20]. Approximately 100 μg of metagenomic DNA was run on a preparative pulsed-field gel (Bio-Rad CHEF DR®III; 0.1-40 s switch time, 6 V/cm, 0.5 × TBE buffer, 120° included angle, 16 h), and the appropriate size of DNA ranging from 30-50 kb was isolated, electroeluted, and dialyzed against 0.5 × TE buffer for further Fosmid library construction. The purified DNA fragments were end-repaired by End-repaired enzyme mix. After size fractionation and purification, the blunt-ended, 5'-phosphorylated DNA was ligated into the cloning-ready Copycontrol pCC2FOS vector, and the recombinant molecules were packaged *in vitro *with a MaxPlaxTM Lambda packaging kit (Epicentre Biotechnologies, Madison, Wisconsin, USA). The selected unique fosmid clone was named FosB12L1 (showing strong lipolytic activity on tributyrin plate), and purified, partially digested with *Sau *3AI to obtain 3-5 kb size DNA, and ligated into a purified *Bam *HI/BAP pUC118 vector from Takara. Ligation products were transformed into *E. coli *TOP10 cells (Tiangen) and spread out on LB (ampicillin, 100 μg/ml) plates containing 1% (v/v) tributyrin as the indicator substrate [21].

### Identification of lipolytic clones and DNA sequence analysis

The DNA fragment obtained was sequenced with primer walking method by SinoGenoMax Co. Ltd (Chinese National Human Genome Center, Beijing). The ORFs were analyzed using DNAstar (Lynnon Biosoft) and GeneTool software (Syngene), Database searches were performed with the BLAST program via GenomeNet World Wide Web server. Peptide sequences of various enzymes or subunits were extracted from National Center for Biotechnology Information (Washington, D.C).

### Phylogenetic analysis

Deduced amino acid sequences of 8 lipolytic enzymes were subjected to protein phylogenetic analysis. Sequence alignment was performed by using CLUSTAL_W program [23] and visually examined with BoxShade Server program. Phylogenetic tree was generated using the neighbor joining method of Saitou and Nei [22] with MEGA 4.0 software [24].

### Protein expression and purification

For the overexpression of EstAS, the full length of the *estAS *gene was amplified using primers EstAS-f and EstAS-r (Table 2) and high fidelity PrimeSTAR™HS DNA Polymerase (code: DR010SA, Takara). The primer pairs with restriction enzyme sites (underlined) for *Hind *III and *Nde *I were designed to generate an N-terminal His-tag of the recombinant esterase. The integrity of the nucleotide sequence of all newly constructed plasmids was confirmed by DNA sequencing. The *EstAS *gene was cloned into an expression vector, pET28a(+), and the recombinant plasmid p*EstAS*-His was transformed into *E. coli *BL21 (DE3) cells. When the cell density at 600 nm reached around 0.6, 1 mM isopropylthio-β-D-galactoside was added for the induction, following a further cultivation for 4 h at 30°C. Then cells were harvested by centrifugation, resuspended in a 50 mM sodium phosphate buffer (pH 8.0) containing 10 mM imidazole, and disrupted by sonication. The protein was applied to metal-chelating chromatography using Ni-NTA affinity chromatography (Novagen) according to the manufacturer's instructions. SDS polyacrylamide gel electrophoresis was carried out according to Sambrook and Russell [17].

**Table 2:** Primers used in the study

Primer	Sequence 5'-3'	Description
HTFP061	GTACAACGACACCTAGAC	Sequencing primer for pCC2FOS™
HTRP062	CAGGAAACAGCCTAGGAA	Sequencing primer for pCC2FOS™
M13 primer RV'	CAGGAAACAGCTATGAC	Sequencing primer for pUC118
M13 primer M2	AGCTGTTCACCGAAGTGCTG	Sequencing primer for pUC118
*EstAS *-W1F	GGCGTCGACCGGGTGGAGGA	Genomic walking primer for *EstAS *gene
*EstAS *-W2F	CCCGAGATCCGAGGCGAACT	Genomic walking primer for *EstAS *gene
*EstAS *-W3F	TCTCGAGCACGCCCTTGAAG	Genomic walking primer for *EstAS *gene
*EstAS *-W4F	CGAGTGATAGACGCGATGCC	Genomic walking primer for *EstAS *gene
*EstAS *-f	TCAGCCAT**ATG**TCTTACCCGATCGTCCTGG	Forward primer for *EstAS *gene
	*Nde*I	
*EstAS *-r	CCCAAGCTTCTACGGCAGCTCCGCCGCG	Reverse primer for *EstAS *gene
	*Hin*dIII	

The *Nde*I and *Hin*dIII sites are underlined. The start codon is in bold.

### Characterization and biochemical properties of EstAS

The substrate specificity of the purified enzyme was analyzed using the following substrates of *p*-NP-fatty acyl esters [21,25]: acetate (C2), butyrate (C4), hexanoate (C6), caprylate (C8), decanonate (C10), laurate (C12), myristate (C14) and palmitate (C16). The enzyme was incubated with the ester derivatives (0.5 mM) in 5 ml Tris-HCl buffer (50 mM, pH 8.0) at 40°C for 10 min. The reaction was quenched by adding 5 ml trichloroacetic acid (0.5 mM) and then recovered the original pH value with 5.15 ml NaOH (0.5 mM), and the amount of released *p*-NP was determined by an absorption increase at 405 nm against an enzyme-free blank on a Biospec-1601 spectrophotometer [26,27]. One unit of esterase is defined as the amount needed to release 1 μmol *p*-NP per min under the above conditions. The highest enzyme activity on a substrate (i. e. *p*-NP-hexanoate) was defined as 100%. To determine the presence of esterase activity, the triglyceride derivative 1,2-di-*O*-lauryl-*rac*-glycero-3-glutaric acid 6'-methylresorufin ester (DGGR) (Sigma Aldrich) was used as a chromogenic substrate, and the formation of methyresorufin was analyzed spectrophotometrically at 580 nm [1,28,29]. *Candida rugosa *lipase (Sigma Aldrich) was used as a positive control.

Using *p*-NP-hexanoate (0.5 mM) as substrate, the optimal temperature and pH of purified EstAS was determined, by measuring the enzyme activity after incubation at various temperatures (10-65°C) in 50 mM Tris-HCl buffer (pH 8.0) or after incubation at 35°C for 10 min in the following buffers: 50 mM phosphate buffer (pH 5.0-7.5), 50 mM Tris-HCl (pH 8.0-10.5).

Various metal ions (CoCl_2_, CaCl_2_, ZnCl_2_, MgCl_2_, K_2_SO_4_, FeSO_4_, CuSO_4_, Ni(NO_3_)_2 _and MnSO_4_), and chelating agent EDTA at final concentration of 1 mM were added to the enzyme in 50 mM Tris-HCl (pH 8.0), then assayed for esterase activity after preincubation at 35°C. Effect of detergents or reductors on esterase activity was determined by incubating the enzyme for 30 min at 35°C in 50 mM Tris-HCl (pH 8.0), containing (1%, v/v) Triton X-100, Tween 20 and 80, β-mercaptoethanol, 1, 4-dithiothreitol (DTT), sodium dodecyl sulfate (SDS), cetyltrimethyl ammonium bromide (CTAB), phenylmethanesulfonyl fluoride (PMSF) and diethypyrocarbonate (DEPC), respectively. The enzyme activity without metal ions and detergents was defined as 100%.

### Nucleotide sequence accession number

The DNA sequence of *EstAS *was deposited in DDBJ/EMBL/GenBank under accession number of FJ386490.

## Results and discussion

### Construction of a metagenomic library and screening

About 100 μg DNA was extracted from 1 g activated sludge (wet-weight), and 1.5 μg of size-selected, pulse-field gel-purified high-molecular-weight (HMW) DNA suitable for fosmid cloning was obtained. 300 ng of 30-45 kb purified metagenomic DNA was ligated into the copy control pCC2FOS vector and transfected into *E. coli *EPI300-T1R, producing a metagenomic library of more than 100, 000 fosmids with insert sizes ranging from 28 kb to 40 kb (average size of 35 kb), covering approximately 3.0 Gbp of the total metagenomic DNA. The prokaryotic origin of the library was confirmed by end-sequencing of randomly selected fosmids and comparison with known ORFs in NCBI. Expression screening of the fosmid library based on the hydrolysis of emulsified tributyrin (1%) resulted in the detection of a recombinant clone, FosB12L1, forming a clear zone on the indicator plate.

### Subcloning and identification of the esterase

**Figure 1:**
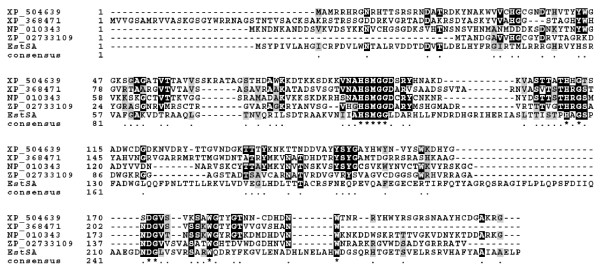
**Conserved sequence blocks from multiple sequence alignment of EstAS from activated sludge metagenomic library and other related proteins**. Sequence alignment was carried out with CLUSTAL_W [24] and BoxShade Server http://www.ch.embnet.org/software/BOX_form.html. XP_504639, esterase/lipase from *Yarrowia lipolytica *CLIB122; XP_368471, LipA from *Magnaporthe grisea *70-15; NP_010343, esterase/lipase from *Saccharomyces cerevisiae *Tg12p; ZP_02733109, lipase from *Gemmata obscuriglobus *UQM 2246.

**Figure 2:**
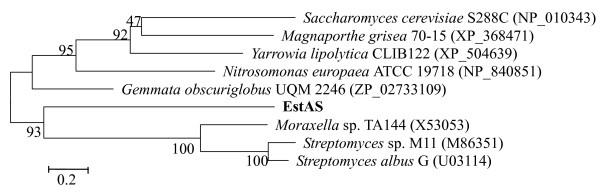
**Phylogenetic analysis of EstAS and closely related proteins**. Phylogenetic analysis was performed using the program MEGA 4.0. Except for EstAS, the protein sequences for previously bacterial lipolytic enzymes were retrieved from GenBank http://www.ncbi.nlm.nih.gov. The numbers at node indicate the bootstrap percentages of 1000 resamples.

The DNA insert (36 kb) of fosmid B12L1 was partial digested by *Sau *3AI and subcloned into pUC118, producing a subclone library of more than 3,000 clones with an average insert size of 3-5 kb. 300 subclones were screened for lipolytic activity. One subclone expressing extracellular lipase/esterase activity was sequenced and assembled into a contig of 3780 bp (data not shown). An ORF of 834 bp encoding a putative lipase/esterase (named EstAS) of 277 amino acid residuals was identified. Amino acid sequence alignment indicated that this EstAS showed quite low identity with other esterase/lipases, highest with the esterase/lipase from *Gemmata obscuriglobus *UQM 2246 (ZP_02733109, 33% identity), followed by the lipase from *Yarrowia lipolytica *CLIB122 (XP_504639, 31% identity), the putative lipase/esterase from *Magnaporthe grisea *70-15 and *Saccharomyces cerevisiase *Tg12p (XP_368471, 31% identity; and NP_010343, 29% identity, respectively), members of the family of fungal hydrolases. And also, the EstAS contained a catalytic triad (Ser92, His125, and Asp249) and a LHYFRG conserved motif (starting from His36), as shown in Fig. 1, which is in close proximity to the active site contributing to the formation of the oxyanion hole that is likely to participate directly in the catalytic process [2,30,31]. Furthermore, to clarify the phylogenetic relationship of the EstAS with other esterases or lipases, a neighbour joining tree was constructed using the amino acid sequence, as shown in Fig. 2. In this tree, EstAS is located closest to the branch of esterase/lipase (accession number X53053) of strain *Moraxella *sp. TA144, and also *Streptomyces *sp. M11, *Streptomyces albus *G (accession numbers M86351 and U03114, respectively), which constitute family III lipases. This result might suggest that the EstAS is a new member of family III lipases.

### Expression and purification of recombinant EstAS

**Figure 3:**
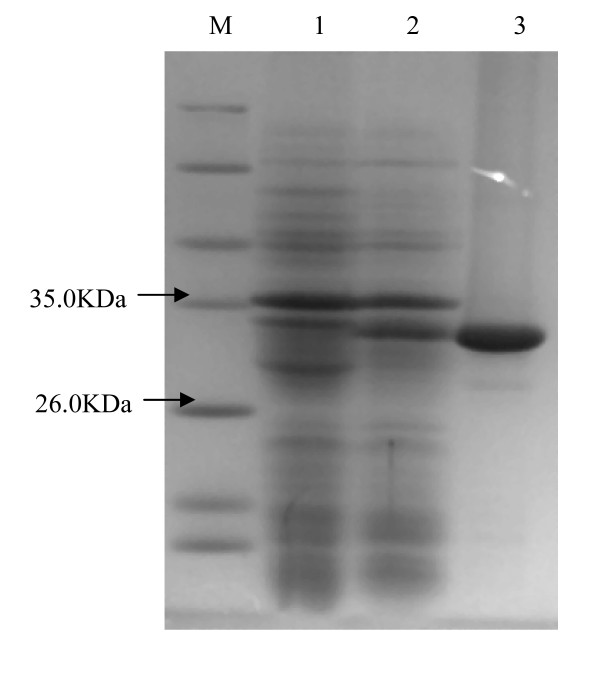
**SDS-PAGE of overexpressed esterase EstAS in *E. coli***. Lane 1: molecular weight protein marker (Tiangen, Cat. No: MP102); lane 2, *E. coli*/pET28a: total protein extract, as negative control; lane 3: induced culture of *E. coli*/pEstAS-His, total protein extract; lane 4: purified EstAS (31 kDa).

To investigate the property of this EstAS, *EstAS *gene was expressed as an N-terminal His-tag fusion protein using pET-28a(+) expression system in *E. coli *BL21(DE3). SDS-PAGE analysis of the purified EstAS showed a single band corresponding to about 31 kDa (Fig. 3), quite agreement with the predicted full length of EstAS. The purity of the purified protein was more than 98% according to SDS-PAGE analysis.

### Substrate specificity of EstAS

**Figure 4:**
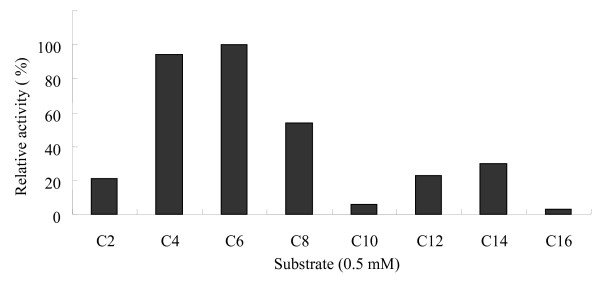
**Substrate specificity of overexpressed and purified esterase**. Relative activity was shown as the percentage of the activity towards 4-nitrophenyl hexanoate. All measurements were done in triplicate. C2, *p*-NP acetate; C4, *p*-NP butyrate; C6, *p*-NP hexanoate; C8, *p*-NP caprylate; C10, *p*-NP decanoate; C12, *p*-NP laurate; C14, *p*-NP myristate and C16, *p*-NP palmitate.

We expressed EstAS as a hexahistidine-tagged (His-tagged) protein and investigated its chain length specificity using *p*-nitrophenyl esters (Sigma). EstAS showed high activity towards short-chain fatty acids (C4, C6 and C8), while much lower towards long-chain fatty acids (>C8) (Fig. 4). In addition, EstAS showed no fluorescence on olive oil plates with rhodamine B. Moreover, the EstAS was not able to hydrolyse DGGR (data not shown), while the lipase from *Candida rugosa *(used as a positive control) was able to hydrolyse DGGR to form chromogenic product, methylresorufin. These results indicate that EstAS is an esterase but a lipase [1,25,32,33].

### Effect of temperature and pH on EstAS

**Figure 5:**
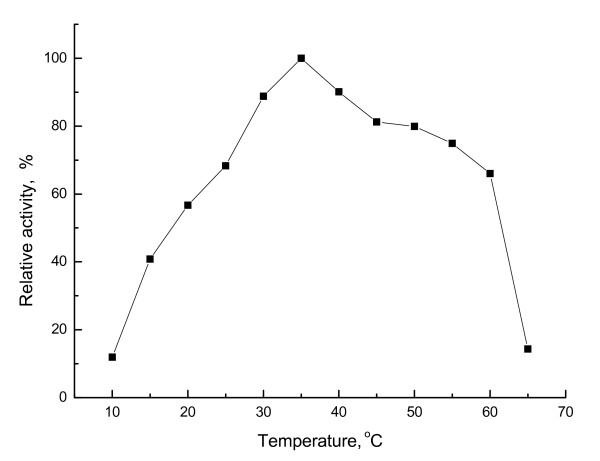
**Apparent temperature optimum of esterase EstAS**. Relative activity of *p*-NP-hexanoate hydrolysis at different temperatures by purified EstAS. The activity was determined at different temperatures at pH 8.0 in 50 mM Tris-HCl buffer. The activity at 35°C was set as 100% (4760 U/ml). All measurements were done in triplicate.

**Figure 6:**
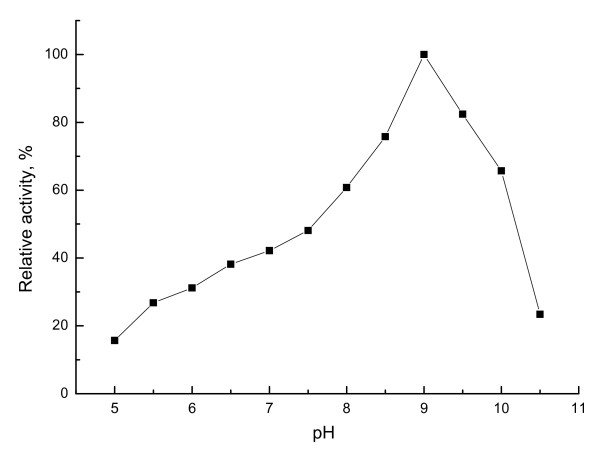
**Effect of pH on the purified esterae EstAS**. Relative activity of *p*-NP-hexanoate hydrolysis was performed in various pH buffers at 35°C (pH 5.0-7.5, 50 mM phosphate buffer; pH 8.0-10.0, 50 mM Tris-HCl buffer). The activity at pH 9.0 was set as 100% (4917 U/ml). All measurements were done in triplicate.

Purified esterase EstAS showed a broader temperature range (optimum at 35°C) than other original esterases, and retained over 65% activity at 60°C (Fig. 5). The esterase YlLip2 from *Yarrowia lipolytica *showed an optimum temperature at 40°C [34], however, it showed a poor thermostability since it lost activity just only incubation at 45°C for 4h. And also, the esterase EstAS showed activity in a rather broad pH range of 5.5-10.5. Maximal activity was observed at pH 9.0 and nearly 23% was still left at pH 10.5 (Fig. 6).

### Effect of metal ions on esterase EstAS

The effect of metal ions on esterase EstAS activity is depicted in Table 3. Among metal ions tested, the activity was slightly increased by Co^2+ ^(117%), Zn^2+ ^(114%) and Fe^2+ ^(103%), and strongly promoted by 1 mM Mn^2+ ^(190%), in comparison with the control. However, it was a bit inhibited by Mg^2+ ^and Ni^2+ ^and almost totally inhibited by Cu^2+ ^(7% residual activity). The fact that its activity was not affected by the chelating agent EDTA might suggest that this esterase is not a metalloenzyme. These results indicated that divalent metal ions, especially Mn^2+^, are necessary for the catalytic activity of esterase EstAS, similarly to metagenomic lipase LipG [35] and esterase EstA from marine metagenome [36]. Therefore, manganese ions might carry out three distinct roles in esterase action: removal of fatty acids as insoluble Mn^2+ ^salts in certain cases, direct enzyme activation acting as cofactor, and stabilizing effect on the enzyme.

**Table 3:** Effect of metal ion on esterase activity

Compounds	Concentration (mM)	Relative activity (%)
Control	0	100.0 ± 3.7
CoCl_2_	1	117.8 ± 2.1
CaCl_2_	1	100.5 ± 3.4
ZnCl_2_	1	114.7 ± 1.3
MgCl_2_	1	81.7 ± 2.9
K_2_SO_4_	1	101 ± 4.1
FeSO_4_	1	103.8 ± 1.6
CuSO_4_	1	7.8 ± 2.3
MnSO_4_	1	192.9 ± 3.8
Ni(NO_3_)_2_	1	46.2 ± 5.2
EDTA	1	121.7 ± 1.2

Activity without metal ions was set as 100% (5370 U/ml). All measurements were done in triplicate.

### Effect of detergents and reductors on esterase EstAS

The effects of detergents and reductors on esterase activity are shown in Table 4. A significant increase in lipolytic activity was observed with addition of 0.1% Tween 80 (128%), Tween 20 (135%), and 1 mM CTAB (138%), Triton X-100 (119%), after 0.5 h preincubation at 35°C. 1 mM β-mercaptoethanl, DTT did not affect the lipolytic activity (102% and 101%, respectively). However, DEPC and SDS had a strong inhibitory effect. In accordance with the esterase reported by Nawani et al. [36], a total loss of activity in the presence of SDS but an enhanced activity in the presence of Triton X-100, and Tween 20 and 80. Interesting, the esterase EstAS activity was not affected by 1 mM PMSF, suggesting it may possess a lid structure, which could eliminate the inhibition effect of PMSF, as some other esterases [10,37,38] and site-directed mutagenesis of amino acid Ser92 will be carried out to confirm the function of Ser92.

**Table 4:** Effect of detergents and enzyme inhibitors on esterase activity

Compounds	Concentration	Relative activity (%)
Control	0	100.0 ± 2.1
β-mercaptoethanl	1 mM	102.7 ± 2.7
DTT	1 mM	101.9 ± 1.9
SDS	1 mM	16.2 ± 9.3
Triton X-100	0.1%	119.6 ± 4.6
Tween 80	0.1%	128.9 ± 0.8
Tween 20	0.1%	135.8 ± 3.1
CTAB	1 mM	138.3 ± 2.1
PMSF	1 mM	100.3 ± 5.2
DEPC	1 mM	48.6 ± 0.7

Activity without detergents and enzyme inhibitors was set as 100% (5290 U/ml). All measurements were done in triplicate.

## Conclusion

In conclusion, we identified a new esterase EstAS belonging to family III lipases from SBR activated sludge metagenomic library. EstAS is a very interesting enzyme with high potential for downstream biotechnological applications. This was confirmed by extensive biochemical characterization, substrate specificity, stability towards addictives including metal ions and detergents, and also, wide pH and temperature spectra. This study also demonstrated that the metagenomic approach is very useful for expanding our knowledge of enzyme diversity, especially for bacterial esterases. Accessing the metagenomic pool of lipases and esterases can be an immediate source of novel biocatalysts, or yield enzymes that can be further specialized by directed evolution.

## Competing interests

The authors declare that they have no competing interests.

## Authors' contributions

TZ participated in the design of experiments, and carried out the study and drafted the manuscript. WJH carried out the SDS-PAGE experiment and sequence alignment. ZPL conceived the study, and participated in its design and coordination and helped to draft the manuscript. All authors read and approved the final manuscript.

## References

[B1] Jaeger KE Dijkstra BW Reetz MT Bacterial biocatalysts: molecular biology, three-dimensional structures, and biotechnological applications of lipases Annu Rev Microbiol 1999 53 315 351 10.1146/annurev.micro.53.1.315 10547694

[B2] Jaeger KE Ransac S Dijkstra BW Colson C Heuvel M van Misset O Bacterial lipases FEMS Microbiol Rev 1994 15 1 29 63 10.1111/j.1574-6976.1994.tb00121.x 7946464

[B3] Schmid A Dordick JS Hauer B Kiener A Wubbolts M Witholt B Industrial biocatalysis today and tomorrow Nature 2001 409 6817 258 268 10.1038/35051736 11196655

[B4] Straathof AJ Panke S Schmid A The production of fine chemicals by biotransformations Curr Opin Biotechnol 2002 13 6 548 556 10.1016/S0958-1669(02)00360-9 12482513

[B5] Handelsman J Metagenomics: Application of genomics to uncultured microorganisms Microbiol Mol Biol Rev 2004 68 4 669 685 10.1128/MMBR.68.4.669-685.2004 15590779 PMC539003

[B6] Lee SW Won K Lim HK Kim JC Choi GJ Cho KY Screening for novel lipolytic enzymes from uncultured soil microorganisms Appl Microbiol Biotechnol 2004 65 6 720 726 10.1007/s00253-004-1722-3 15365646

[B7] Elend C Schmeisser C Leggewie C Babiak P Carballeira JD Steele HL Reymond JL Jaeger KE Streit WR Isolation and biochemical characterization of two novel metagenome-derived esterases Appl Environ Microbiol 2006 72 5 3637 3645 10.1128/AEM.72.5.3637-3645.2006 16672512 PMC1472341

[B8] Henne A Schmitz RA Bomeke M Gottschalk G Daniel R Screening of environmental DNA libraries for the presence of genes conferring lipolytic activity on Escherichia coli Appl Environ Microbiol 2000 66 7 3113 3116 10.1128/AEM.66.7.3113-3116.2000 10877816 PMC92121

[B9] Li G Wang K Liu YH Molecular cloning and characterization of a novel pyrethroid-hydrolyzing esterase originating from the metagenome Microb Cell Fact 2008 7 38 19116015 10.1186/1475-2859-7-38 PMC2657102

[B10] Chu XM He HZ Guo CQ Sun BL Identification of two novel esterases from a marine metagenomic library derived from South China Sea Appl Microbiol Biotechnol 2008 80 4 615 625 10.1007/s00253-008-1566-3 18600322

[B11] Jeon JH Kim JT Kim YJ Kim HK Lee HS Kang SG Kim SJ Lee JH Cloning and characterization of a new cold-active lipase from a deep-sea sediment metagenome Appl Microbiol Biotechnol 2009 81 5 865 874 10.1007/s00253-008-1656-2 18773201

[B12] Park HJ Jeon JH Kang SG Lee JH Lee SA Kim HK Functional expression and refolding of new alkaline esterase, EM2L8 from deep-sea sediment metagenome Prot Expr Purif 2007 52 2 340 347 10.1016/j.pep.2006.10.010 17126562

[B13] Ranjan R Grover A Kapardar RK Sharma R Isolation of novel lipolytic genes from uncultured bacteria of pond water Biochem Biophys Res Commun 2005 335 1 57 65 10.1016/j.bbrc.2005.07.046 16054111

[B14] Rees HC Grant S Jones B Grant WD Heaphy S Detecting cellulase and esterase enzyme activities encoded by novel genes present in environmental DNA libraries Extremophiles 2003 7 5 415 421 10.1007/s00792-003-0339-2 12845554

[B15] Rhee JK Ahn DG Kim YG Oh JW New thermophilic and thermostable esterase with sequence similarity to the hormone-sensitive lipase family, cloned from a metagenomic library Appl Environ Microbiol 2005 71 2 817 825 10.1128/AEM.71.2.817-825.2005 15691936 PMC546692

[B16] Wu C Sun BL Identification of novel esterase from metagenomic library of Yangtze River J Microbiol Biotechnol 2009 19 2 187 193 10.4014/jmb.0804.292 19307769

[B17] Sambrook J Russell DW Molecular cloning: a laboratory manual 2001 Cold Spring Harbor Laboratory Press, New York

[B18] Birnboim HC Doly J A rapid alkaline extraction procedure for screening recombinant plasmid DNA Nucleic Acids Res 1979 7 6 1513 1523 10.1093/nar/7.6.1513 388356 PMC342324

[B19] Zhou J Bruns MA Tiedje JM DNA recovery from soils of diverse composition Appl Environ Microbiol 1996 62 2 316 322 8593035 10.1128/aem.62.2.316-322.1996 PMC167800

[B20] Xi F Fu LY Wang GZ Zheng TL A simple method for removing humic acids from marine sediment samples prior to DNA extraction Chin High Technol Lett 2006 16 5 539 544

[B21] Roh C Villatte F Isolation of a low-temperature adapted lipolytic enzyme from uncultivated microorganism J Appl Microbiol 2008 105 1 116 123 10.1111/j.1365-2672.2007.03717.x 18248379

[B22] Saitou N Nei M The neighbor-joining method: a new method for reconstructing phylogenetic trees Mol Biol Evol 1987 4 4 406 425 3447015 10.1093/oxfordjournals.molbev.a040454

[B23] Thompson JD Gibson TJ Plewniak F Jeanmougin F Higgins DG The CLUSTAL_X windows interface: flexible strategies for multiple sequence alignment aided by quality analysis tools Nucleic Acids Res 1997 25 24 4876 4882 10.1093/nar/25.24.4876 9396791 PMC147148

[B24] Tamura K Dudley J Nei M Kumar S MEGA4: Molecular Evolutionary Genetics Analysis (MEGA) software version 4.0 Mol Biol Evol 2007 24 8 1596 1599 10.1093/molbev/msm092 17488738

[B25] Hardeman F Sjoling S Metagenomic approach for the isolation of a novel low-temperature-active lipase from uncultured bacteria of marine sediment FEMS Microbiol Ecol 2007 59 2 524 534 10.1111/j.1574-6941.2006.00206.x 17328767

[B26] Jiang HF Wang YQ Liu CG Comparison and improvement of three determination methods for lipase activity Chem Eng 2007 24 8 72 75

[B27] Pignede G Wang HJ Fudalej F Gaillardin C Seman M Nicaud JM Characterization of an extracellular lipase encoded by LIP2 in Yarrowia lipolytica J Bacteriol 2000 182 10 2802 2810 10.1128/JB.182.10.2802-2810.2000 10781549 PMC101989

[B28] Panteghini M Bonora R Pagani F Measurement of pancreatic lipase activity in serum by a kinetic colorimetric assay using a new chromogenic substrate Ann Clin Biochem 2001 38 4 365 370 10.1258/0004563011900876 11471878

[B29] Zandonella G Haalck L Spener F Faber K Paltauf F Hermetter A Enantiomeric perylene-glycerolipids as fluorogenic substrates for a dual wavelength assay of lipase activity and stereoselectivity Chirality 1996 8 7 481 489 10.1002/(SICI)1520-636X(1996)8:7<481::AID-CHIR4>3.0.CO;2-E 8970745

[B30] Ollis DL Cheah E Cygler M Dijkstra B Frolow F Franken SM Harel M Remington SJ Silman I Schrag J Sussman JL Verschueren KHG Goldman A The alpha/beta-hydrolase Fold Protein Eng 1992 5 3 197 211 10.1093/protein/5.3.197 1409539

[B31] Bell PJL Sunna A Gibbs MD Curach NC Nevalainen H Bergquist PL Prospecting for novel lipase genes using PCR Microbiology-Sgm 2002 148 8 2283 2291 10.1099/00221287-148-8-2283 12177322

[B32] Verger R Interfacial activation of lipases: Facts and artifacts Trends Biotechnol 1997 15 1 32 38 10.1016/S0167-7799(96)10064-0

[B33] Jaeger KE Eggert T Lipases for biotechnology Curr Opin Biotechnol 2002 13 4 390 397 10.1016/S0958-1669(02)00341-5 12323363

[B34] Yu MR Qin SW Tan TW Purification and characterization of the extracellular lipase Lip2 from Yarrowia lipolytica Process Biochem 2007 42 3 384 391 10.1016/j.procbio.2006.09.019

[B35] Lee MH Lee CH Oh TK Song JK Yoon JH Isolation and characterization of a novel lipase from a metagenomic library of tidal flat sediments: evidence for a new family of bacterial lipases Appl Environ Microbiol 2006 72 11 7406 7409 10.1128/AEM.01157-06 16950897 PMC1636159

[B36] Nawani N Dosanjh NS Kaur J A novel thermostable lipase from a thermophilic *Bacillus *sp.: Characterization and esterification studies Biotechnol Lett 1998 20 10 997 1000 10.1023/A:1005430215849

[B37] De Simone G Menchise V Manco G Mandrich L Sorrentino N Lang D Rossi M Pedone C The crystal structure of a hyper-thermophilic carboxylesterase from the archaeon *Archaeoglobus fulgidus* J Mol Biol 2001 314 3 507 518 10.1006/jmbi.2001.5152 11846563

[B38] De Simone G Galdiero S Manco G Lang D Rossi M Pedone C A snapshot of a transition state analogue of a novel thermophilic esterase belonging to the subfamily of mammalian hormone-sensitive lipase J Mol Biol 2000 303 5 385 10.1006/jmbi.2000.4195 11061974

