# Gene cloning and characterization of a novel esterase from activated sludge metagenome

**DOI:** 10.1186/1475-2859-9-25

**Published:** 2010-04-28

**Authors:** Tao Zhang, WenJun Han

**Affiliations:** 1 State Key Laboratory of Microbial Resources, Institute of Microbiology, Chinese Academy of Sciences, Beijing 100101, PR China; 2 School of Life Sciences and Biotechnology, Mianyang Normal University, Mianyang 621000, PR China

## 

This article [1] was submitted and published without the permission of all original authors. An amended author list has been provided in this correction article and the Authors' contributions and Competing interests section modified accordingly. Mr Tao Zhang apologies to the other authors for this oversight.

Additionally figure three in the original article was completed by another colleague, is of a different protein to that stated and was used without his permission. A correct figure completed by Mr Tao Zhang of the correct protein can be found in this correction article (Fig 1).

**Figure 1:**
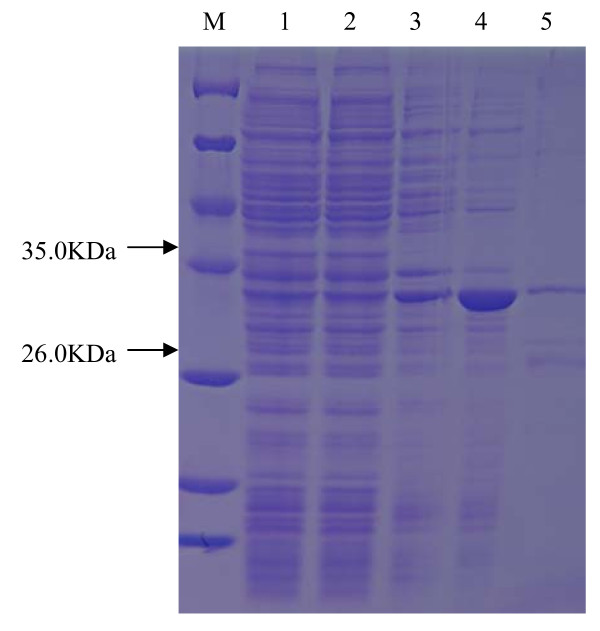
**SDS-PAGE of overexpressed esterase EstAS in *E. coli***. Lane 1: molecular weight protein marker (Tiangen, Cat. No: MP102); lane 2, *E. coli*/pET28a: total protein extract, as negative control; lane 3: induced culture of *E. coli*/pET28a, as negative control; lane 4: total protein extract *E. coli*/pEstAS-His; lane 5, total protein extract, induced culture of *E. coli*/pEstAS-His; lane 6: purified EstAS (31 kDa).

Mr Tao Zhang's institution has investigated this incident of author misconduct.

## Competing interests

The authors declare that they have no competing interests.

## Authors' contributions

TZ contributed to the construction of metagenomic DNA library, esterese gene screening, enzyme characteristics analysis and manuscript drafting and revision.

WJH contributed to esterase gene cloning, EstAS expression vector construction and resequencing, data collection and manuscript revision.
